# GnRH antagonist-induced ovarian suppression does not protect the ovarian reserve from cyclophosphamide chemotherapy in tumour-bearing mice

**DOI:** 10.3389/fendo.2026.1959150

**Published:** 2026-10-27

**Authors:** Yasmin M Lewis, Daniella Herszberg, Kate Fowler, Michael Hun, Emmaline Brown, Sherene Loi, Amy L Winship, Karla J Hutt

**Affiliations:** 1 Development and Stem Cell Program, Department of Anatomy and Developmental Biology, Biomedicine Discovery Institute, Monash University, Clayton, VIC, Australia; 2 Division of Research, Peter MacCallum Cancer Centre, University of Melbourne, Melbourne, VIC, Australia; 3 Sir Peter MacCallum Department of Oncology, University of Melbourne, Melbourne, VIC, Australia

**Keywords:** cyclophosphamide, degarelix, fertility preservation, gonadotropin-releasing hormone antagonist, ovarian fibrosis, ovarian reserve

## Abstract

**Background:**

Gonadotropin-releasing hormone (GnRH) analogues are used clinically to induce ovarian suppression during chemotherapy treatment and are proposed to protect the ovaries from chemotherapy-induced damage, yet their efficacy and underlying mechanisms remain uncertain. While most preclinical and clinical studies have focused on GnRH agonists, far less is known about the effects of GnRH antagonists in this context, despite their more rapid and direct suppression of gonadotrophin signaling.

**Materials and methods:**

We used the GnRH antagonist degarelix to induce ovarian suppression in an orthotopic tumour-bearing mouse model. We then evaluated whether degarelix protected against cyclophosphamide-induced ovarian damage in reproductively young and aged mice receiving one or two chemotherapy doses. Ovarian function was assessed by quantifying ovarian volume, corpora lutea number (a marker of ovulation), and serum anti-Müllerian hormone (AMH) levels, as well as follicle enumeration and histological assessment of ovarian fibrosis.

**Results:**

Degarelix suppressed ovulatory activity, as indicated by reduced corpora lutea number and ovarian volume. However, degarelix was associated with increased ovarian fibrosis in young mice treated with single-dose cyclophosphamide and failed to protect the ovarian reserve following either single or two doses of cyclophosphamide in both age groups. Moreover, degarelix did not prevent reductions in circulating AMH, with all treatment groups displaying reduced AMH levels compared with controls in young mice.

**Conclusions:**

Together, our findings do not support a protective effect of GnRH antagonist treatment against cyclophosphamide-induced ovarian damage.

## 1 Introduction

Advances in cancer detection and treatment have led to steadily improving survival among adolescents and young adults, shifting attention toward long-term quality of life, including fertility and endocrine health (1, 2). Chemotherapy-induced ovarian toxicity, particularly depletion of the ovarian reserve, increases the risk of premature ovarian insufficiency (POI), defined as depletion of the ovarian reserve and loss of regular ovarian function prior to age 40, as well as early menopause (3–5). These effects have implications for future childbearing but also carry cascading consequences for overall health, including increased risks of cardiovascular disease, osteoporosis and neurological disorders (3, 6). Therefore, strategies that mitigate ovarian damage while maintaining anti-tumour efficacy are a major priority within the field.

The ovarian reserve, established during gestation, represents a female’s non-renewable lifetime supply of oocytes. Protecting the primordial follicles that make up the ovarian reserve is essential for preserving reproductive potential and hormone production (7). However, *in situ* quantification of the ovarian reserve is not possible, as current techniques require histological analysis of the entire ovary (8, 9). In clinical practice, serum anti-Müllerian hormone (AMH) is widely used as an indirect marker of ovarian reserve. However, AMH is produced by granulosa cells of growing pre-antral and small antral follicles, rather than by the quiescent primordial follicle pool, making it an indirect measure (10, 11). Therefore, preclinical animal models are indispensable for quantifying ovarian reserve protection from chemotherapy for fertility preservation.

Alkylating agents, such as cyclophosphamide, are among the most widely used and most ovotoxic chemotherapeutic agents (4, 12, 13). Cyclophosphamide forms DNA crosslinks and DNA double-strand breaks that ultimately trigger cell death (12, 14, 15). Cyclophosphamide can directly damage primordial follicle oocytes, resulting in ovarian reserve depletion. It may also impair granulosa and thecal cell survival, contributing to growing follicle loss and potentially accelerating primordial follicle activation (4, 16). Beyond direct follicle loss, cyclophosphamide also induces ovarian fibrosis, which has been associated with impaired ovarian function (17, 18).

Established fertility preservation options for female patients, including oocyte, embryo and ovarian tissue cryopreservation are invasive, time consuming, and may not be feasible for patients requiring urgent treatment (19). While these approaches have transformed fertility preservation, they do not prevent treatment-induced POI (19, 20). As a result, there is considerable interest in pharmacological co-treatments that can be delivered alongside chemotherapy to reduce ovarian damage. Gonadotropin-releasing hormone (GnRH) analogues, including agonists and antagonists, can be administered concurrently with chemotherapy to potentially mitigate ovarian damage. GnRH agonists are used as a non-invasive, reversible pharmacological adjunct to established fertility preservation strategies such as oocyte and embryo cryopreservation, although they are not considered a substitute for these approaches (21–23). Clinical trials and meta-analyses have reported reduced POI risk and improved pregnancy rates following GnRH analogue co-treatment, particularly in patients with hormone receptor-negative breast cancer. However, interpretation is limited by the use of menstrual resumption as a surrogate endpoint, as well as inconsistent findings across cancer types. Preclinical findings have also been inconsistent (24–28). GnRH analogues act at the pituitary GnRH receptor to suppress gonadotrophin secretion. GnRH agonists induce an initial transient flare in gonadotrophin release followed by sustained suppression, whereas GnRH antagonists produce rapid suppression without a flare phase (29, 30). By inducing a hypoestrogenic state, GnRH analogue treatment is frequently associated with menopausal-like adverse effects such as hot flashes, headaches, vaginal dryness and mood change (31, 32).

Based on observations that POI appears less severe when chemotherapy is administered before puberty, GnRH analogue treatment is thought to mimic a prepubertal hormonal milieu with reduced gonadotropin signaling to the ovaries (33, 34). Several mechanisms have been proposed to explain the potential protective effects of GnRH analogues. These include reduced FSH signaling, which may limit follicle activation and subsequent depletion of the ovarian reserve, and reduced ovarian perfusion during chemotherapy (33, 35–37). However, clinical and experimental evidence supporting these mechanisms remains inconsistent and robust mechanistic studies are limited. Several studies in rodents have reported that GnRH analogues fail to protect the ovarian reserve from cyclophosphamide or cisplatin, while others have reported preserved follicle numbers or AMH following GnRH analogue co-treatment (38–41). Whether gonadotrophin suppression protects the ovary during chemotherapy therefore remains unresolved.

Although GnRH agonists are more commonly used in clinical fertility preservation settings, GnRH antagonists such as degarelix provide rapid and reversible suppression of gonadotrophins and are therefore well suited for tumour-bearing experimental models where timely endocrine modulation is required. GnRH antagonists have received considerably less attention in ovarian protection studies than agonists. Moreover, limited preclinical evidence raises concerns regarding potential antagonist-associated ovarian toxicity. Cetrorelix, for example, has been reported to deplete the ovarian reserve in rats and mice in the absence of chemotherapy (38, 41). In this study, we used degarelix to model acute gonadotrophin suppression to directly investigate the impact of GnRH pathway inhibition on ovarian function during chemotherapy exposure in a clinically relevant tumour-bearing mouse model.

## 2 Methods

### 2.1 Ethics statement

All animal experiments and procedures were approved by the Peter MacCallum Animal Experimentation Ethics Committee and conducted in accordance with the National Health and Medical Research Council (NHMRC) Australian Code for the Care and Use of Animals for Scientific Purposes. Mice were housed in a temperature-controlled facility, under a 12-hour light-dark cycle, with free access to food and water.

### 2.2 Animal treatment

Female, six-week-old (reproductively young), and six-month-old (reproductively aged) C57BL/6J mice were injected orthotopically with AT3-OVA cells into the fourth mammary fat pad as previously described (42). Once tumours reached 30 mm^3^, approximately two weeks following inoculation, mice were randomly allocated into treatment groups. Mice were administered 100 mg/kg of cyclophosphamide (Endoxan, Baxter) intraperitoneally, 78 mg/kg of degarelix (Firmagon, Ferring) subcutaneously or saline vehicle control. Mice allocated to the two-dose cyclophosphamide regimen were subjected to a second dose of cyclophosphamide the following week. The cyclophosphamide dose is standard for murine models of ovarian reserve depletion (43, 44), and the degarelix dose was based on prior use in mice (45). Ovaries and serum were collected from mice when tumour volume reached 1100 mm^3–^1500 mm^3^, which represented the predefined tissue-collection endpoint under the institutional animal ethics approval and was selected in accordance with the approved tumour-monitoring and humane-endpoint criteria. At tissue collection, mice were three to four months of age for the reproductively young cohort and seven to nine months of age for the aged cohort.

### 2.3 Ovarian follicle and corpora lutea quantification

One ovary from each mouse was fixed in 10% (v/v) neutral buffered formalin, embedded in paraffin and exhaustively sectioned at five µm. Every ninth section was stained with Periodic acid–Schiff (PAS) and imaged on the Aperio Digital Pathology Slide Scanner (Leica Biosystems; 20× objective), then visualized by a blinded assessor using Aperio ImageScope software (Leica Biosystems). Ovarian follicles were classified as described (9, 46). Growing follicles were classified as atretic according to established histomorphological criteria, including follicular degeneration and the presence of pyknotic granulosa cells, as previously described (9, 46). Atresia was assessed in every ninth PAS-stained section throughout the ovary. Corpora lutea were counted in every ninth section. Due to their large size and persistence across multiple sections, raw corpora lutea counts were not multiplied by the sampling interval.

### 3.4 Ovarian volume

Ovarian volume is used in clinical practice as an indirect measure of ovarian reserve (47, 48). Ovarian volume was estimated using the Cavalieri principle as described (49). Every ninth PAS-stained section was assessed at 10× objective using Stereo Investigator software (MBF Bioscience) on an Olympus BX50 microscope. The Cavalieri probe was used to superimpose a grid of equally spaced points, 200 µm apart, on each section. Points intersecting the ovary were marked on every ninth section. Software estimates ovarian volume according to the following equation:


$$V=a\left(p\right)\times d\times t\sum_{i=0}^{n}Pi$$

*V* = Estimated volume of ovary.

*a*(*p*) = Area of each grid point.

*d* = Distance between two sequential sections.

*t* = Thickness of each section.

$\sum_{i=0}^{n}Pi$ =Sum of all points marked in the sections (P_1_ + P_2_ + P_3_…).

### 2.5 Picrosirius red staining

One representative section from the middle of the ovary was stained using the Picro Sirius Red Stain Kit (AbCam, #Ab150681) to visualise collagen I and III deposition for each mouse as previously described (49). Stained sections were imaged using the Aperio Digital Pathology Slide Scanner (Leica Biosystems; 20× objective). QuPath was utilised to quantify the area of positive staining for each ovary section. A new threshold was set for each cohort utilising the collagen-rich connective tissue surrounding the ovary. The threshold was applied to all images analysed within the same cohort. Medullary fibrosis was quantified within a defined region measuring 350 µm × 550 µm. This approach excluded large growing follicles and corpora lutea, whose relatively high collagen content could otherwise confound fibrosis measurements.

### 2.6 AMH enzyme linked immunosorbent assay

Serum AMH concentrations in young tumour-bearing mice were measured in duplicate using a mouse AMH ELISA kit (AnshLabs, #AL-113) according to the manufacturer’s instructions. Absorbance was measured on a ClarioStar microplate reader (BMG Labtech).

### 2.7 Statistics

Statistical analysis was performed using GraphPad Prism 10.1.0 Software. Normality was tested using a Shapiro–Wilk normality test. Parametric data were analysed by one-way ANOVA and Šidák’s multiple-comparisons test. Homogeneity of variance was assessed using the Brown-Forsythe test; if variances differed significantly, data were analysed by Brown-Forsythe and Welch’s ANOVA with Dunnett’s T3 *post hoc* test. Non-parametric data were analysed by Kruskal-Wallis and Dunn’s multiple comparisons test. Data are presented as mean ± SEM and considered significant if p< 0.05.

## 3 Results

### 3.1 Effects of degarelix on corpora lutea number and ovarian volume

Reproductively young and aged female C57BL/6J tumour-bearing mice were treated with saline vehicle, degarelix (monthly for the duration of the study), cyclophosphamide (one or two 100 mg/kg i.p. doses administered one week apart), or combination treatment (**Figure 1A**; **Supplementary Table 1**). The number of corpora lutea and ovarian volume were quantified using one ovary from each mouse to assess ovulation and gonadotrophin suppression (**Figure 1B**). Corpora lutea were detected in all saline-treated control ovaries from both young and aged mice, consistent with recent ovulation (**Figures 1C, D, F, G**). Treatment with cyclophosphamide alone did not significantly alter corpora lutea number in either age group following single or two-dose treatment regimens (**Figures 1D, G**). Corpora lutea were absent in all ovaries from mice treated with degarelix alone or in combination with a single dose of cyclophosphamide (young: p=0.02 & p=0.03) however this did not reach statistical significance in aged mice (p=0.06 & p=0.06) (**Figure 1D**). Similar findings were observed in young and aged mice receiving the two-dose cyclophosphamide regimen, with corpora lutea absent in all but one mouse treated with degarelix (young: p=0.01 & p=0.01; aged: p=0.009 & p=0.002) (**Figures 1F, G**). Consistent with the marked reduction or absence of corpora lutea, ovaries from reproductively young and aged mice treated with degarelix (alone or with cyclophosphamide single or two doses) were visually smaller than those from saline and cyclophosphamide alone groups (**Figures 1C, F**). To quantify these observations, ovarian volume was quantified histologically. Treatment with single or two doses of cyclophosphamide alone did not significantly alter ovarian volume in either age group compared to controls (**Figures 1E, H**). Compared with controls, degarelix alone reduced ovarian volume by 62% in young mice (p = 0.009) and 74% in aged mice (p = 0.03). Degarelix combined with single-dose cyclophosphamide reduced ovarian volume by 69% and 77%, respectively (both p = 0.02) (**Figure 1E**). Similarly, ovarian volume was significantly reduced by 68% and 79% in two-dose cyclophosphamide plus degarelix groups compared with cyclophosphamide alone in both age groups respectively (p=0.001 & p<0.0001) (**Figure 1H**). In PAS-stained sections, stromal cells in ovaries from degarelix-treated and degarelix and cyclophosphamide co-treated ovaries appeared morphologically altered, exhibiting an atrophied appearance with darker nuclei and cytoplasm compared with control and cyclophosphamide alone ovaries (**Supplementary Figure 1**).

**Figure 1:**
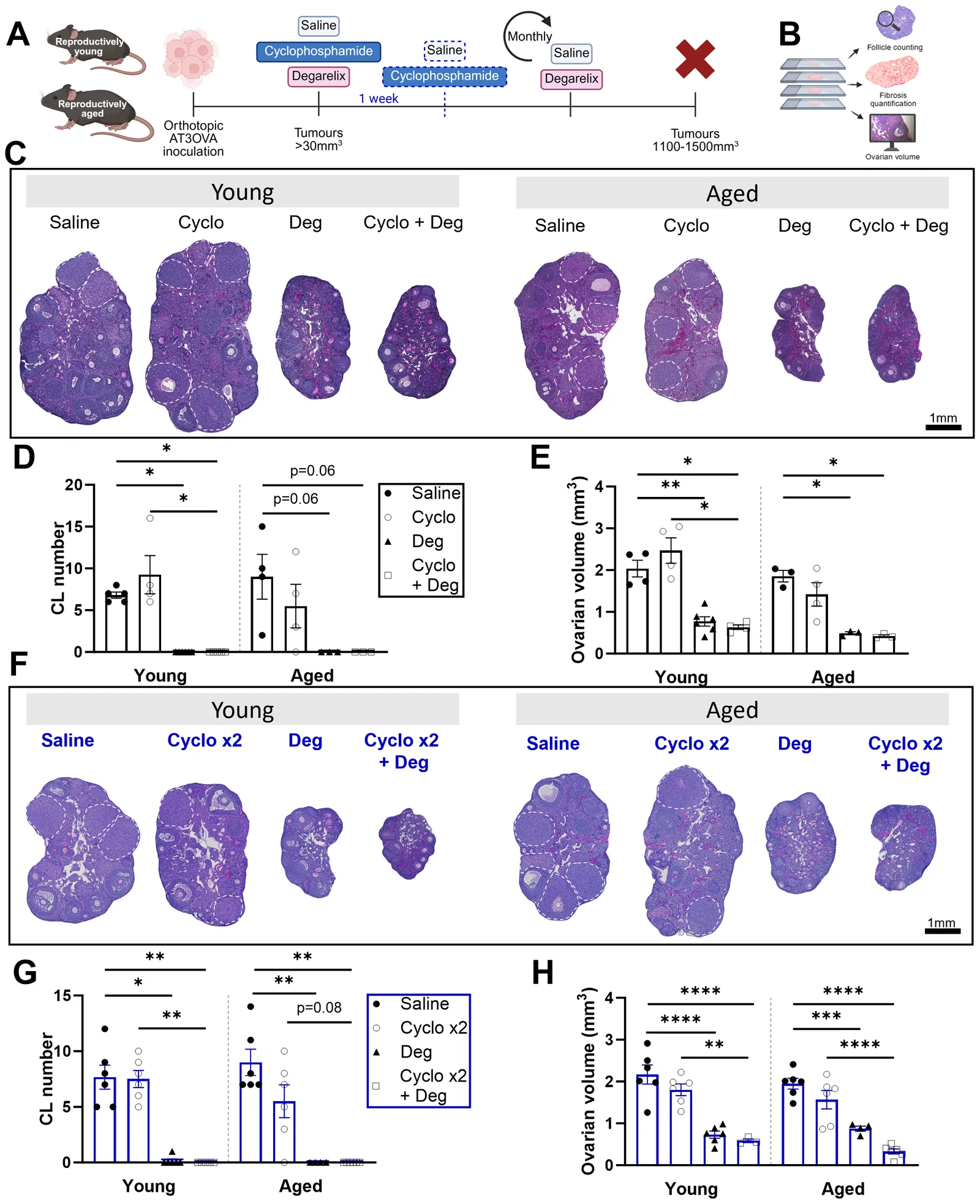
Effects of degarelix and cyclophosphamide on corpora lutea number and ovarian volume in tumour-bearing mice. **(A)** Experimental design. Reproductively young and aged female C57BL/6J mice were inoculated orthotopically with AT3-OVA breast cancer cells. Once tumours reached approximately 30 mm³, mice received saline vehicle, cyclophosphamide (100 mg/kg), degarelix (78 mg/kg) or combination treatment. Mice allocated to the two-dose cyclophosphamide cohort received a second dose one week after the first. Tissues were collected when tumour volumes reached the predefined endpoint of 1,100–1,500 mm³. **(B)** Schematic of ovarian tissue processing for histological analysis. **(C)** Representative periodic acid–Schiff (PAS)-stained ovarian sections from the single-dose cohort, with corpora lutea indicated. Scale bar: 1 mm. **(D, E)** Quantification of corpora lutea number and ovarian volume, respectively, in the single-dose cohort. **(F)** Representative PAS-stained ovarian sections from the two-dose cohort, with corpora lutea indicated. Scale bar: 1 mm. **(G, H)** Quantification of corpora lutea number and ovarian volume, respectively, in the two-dose cohort. Each data point represents one independent animal. Sample sizes for corpora lutea counts in the single-dose cohort were n = 5, 4, 6 and 5 for young saline, cyclophosphamide, degarelix and combination groups, respectively; corresponding aged groups comprised n = 4, 4, 3 and 3. For ovarian volume, the aged saline group comprised n = 3, with the remaining single-dose group sizes unchanged. In the two-dose cohort, corpora lutea counts comprised n = 6, 6, 6 and 5 in young mice and n = 6, 6, 4 and 6 in aged mice. For ovarian volume, the young combination group comprised n = 3, with the remaining sample sizes unchanged. Data are presented as mean ± SEM. All data were tested for normality using Shapiro-Wilk test and homogeneity variance using Brown-Forsythe test. All data were analysed using a one-way ANOVA with Šidák’s multiple-comparisons test, Brown-Forsythe and Welch’s ANOVA with Dunnett’s T3 or Kruskal-Wallis test with Dunn’s multiple comparisons. *p ≤ 0.05, **p ≤ 0.01 ***p ≤ 0.001, ****p ≤ 0.0001.

### 3.2 Assessments of ovarian fibrosis following degarelix and cyclophosphamide treatment

In young tumour-bearing mice, ovarian fibrosis was greater in the degarelix plus single-dose cyclophosphamide group (p=0.03) than in control and cyclophosphamide alone groups (**Figures 2A, B**). In the medulla, fibrosis was greater than in the controls in young, but not aged, single-dose cyclophosphamide and degarelix co-treated mice (p=0.03) (**Figure 2C**). In the two-dose cyclophosphamide cohorts, total ovarian fibrosis was similar between treatment groups (**Figures 2D, E**). Medullary fibrosis was greater in young mice treated with two doses of cyclophosphamide, compared to control. No differences between the remaining treatment groups were observed (**Figure 2F**). No changes in medulla fibrosis were observed between treatments in the aged mice of this cohort (**Figure 2F**).

**Figure 2:**
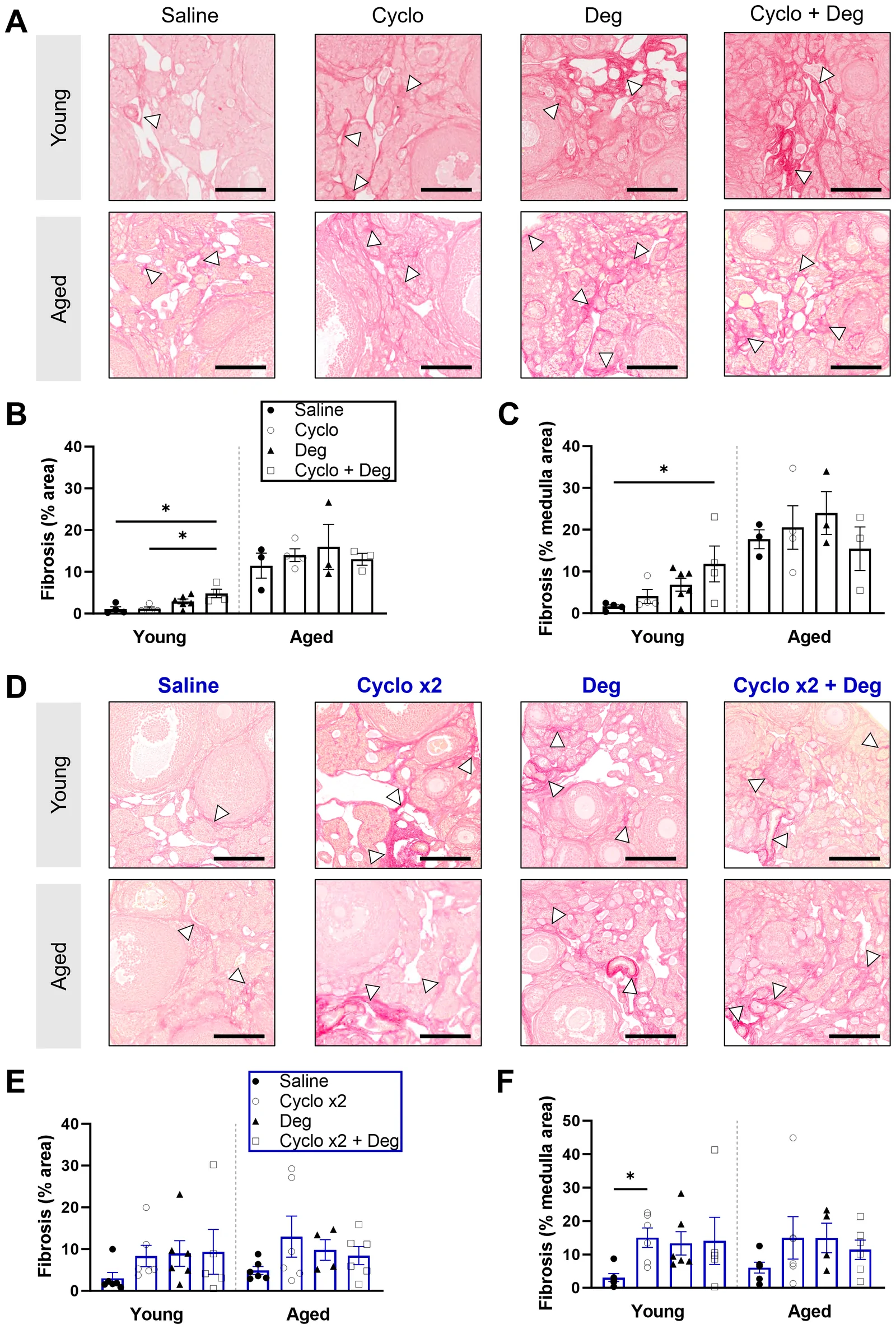
Ovarian collagen deposition following degarelix and cyclophosphamide treatment. **(A)** Representative picrosirius red-stained ovarian sections from the single-dose cyclophosphamide cohort. Arrowheads indicate collagen-positive regions. Scale bar: 500 µm. **(B, C)** Quantification of whole-ovarian and medullary collagen-positive area, respectively, in reproductively young and aged tumour-bearing mice from the single-dose cohort. **(D)** Representative picrosirius red-stained ovarian sections from the two-dose cohort. Arrowheads indicate collagen-positive regions. Scale bar: 500 µm. **(E, F)** Quantification of whole-ovarian and medullary collagen-positive area, respectively, in the two-dose cohort. Each data point represents one independent animal. Sample sizes in the single-dose cohort were n = 5, 4, 6 and 4 for young saline, cyclophosphamide, degarelix and combination groups, respectively. Corresponding aged groups comprised n = 3, 4, 3 and 3. Sample sizes in the two-dose cohort were n = 6, 6, 6 and 5 for young mice and n = 6, 6, 4 and 6 for aged mice. Data are presented as mean ± SEM. All data were tested for normality using Shapiro-Wilk test and homogeneity variance using Brown-Forsythe test. All data were analysed using a one-way ANOVA with Šidák’s multiple-comparisons test, Brown-Forsythe and Welch’s ANOVA with Dunnett’s T3 or Kruskal-Wallis test with Dunn’s multiple comparisons. *p ≤ 0.05.

### 3.3 Impacts of cyclophosphamide with or without degarelix on primordial and primary follicle numbers

Direct follicle counts were conducted for PAS-stained ovary sections to assess the potential of degarelix co-treatment to protect follicles from cyclophosphamide treatment (**Figure 3A**). Single dose cyclophosphamide significantly depleted primordial follicle number by 62% and 89% in young and aged tumour-bearing mice, compared to controls (p=0.02 & p=0.002) (**Figure 3B**). Primordial follicle number was also reduced in cyclophosphamide and degarelix co-treated mice compared with controls (p=0.01 & p=0.03) (**Figure 3B**). In the two-dose cyclophosphamide cohort primordial follicle numbers were also significantly reduced in young animals compared to control, with cyclophosphamide and degarelix co-treatment showing similar reductions to cyclophosphamide alone (p=0.05 & p=0.05) (**Figure 3C****).** Primary follicle number was significantly reduced by 85% in aged tumour-bearing mice treated with single-dose cyclophosphamide compared to controls (p<0.0001); no difference was observed in young mice (**Figures 3D, E**). In aged mice, primary follicle number was also reduced compared with controls following degarelix alone (p=0.005) and cyclophosphamide and degarelix co-treatment (p=0.001) (**Figure 3E**). Two doses of cyclophosphamide reduced primary follicle numbers by 56% in aged mice compared with controls (p = 0.02). Primary follicle numbers were also reduced following co-treatment in young (p = 0.02) and aged (p = 0.006) mice compared with controls (**Figure 3F**).

**Figure 3:**
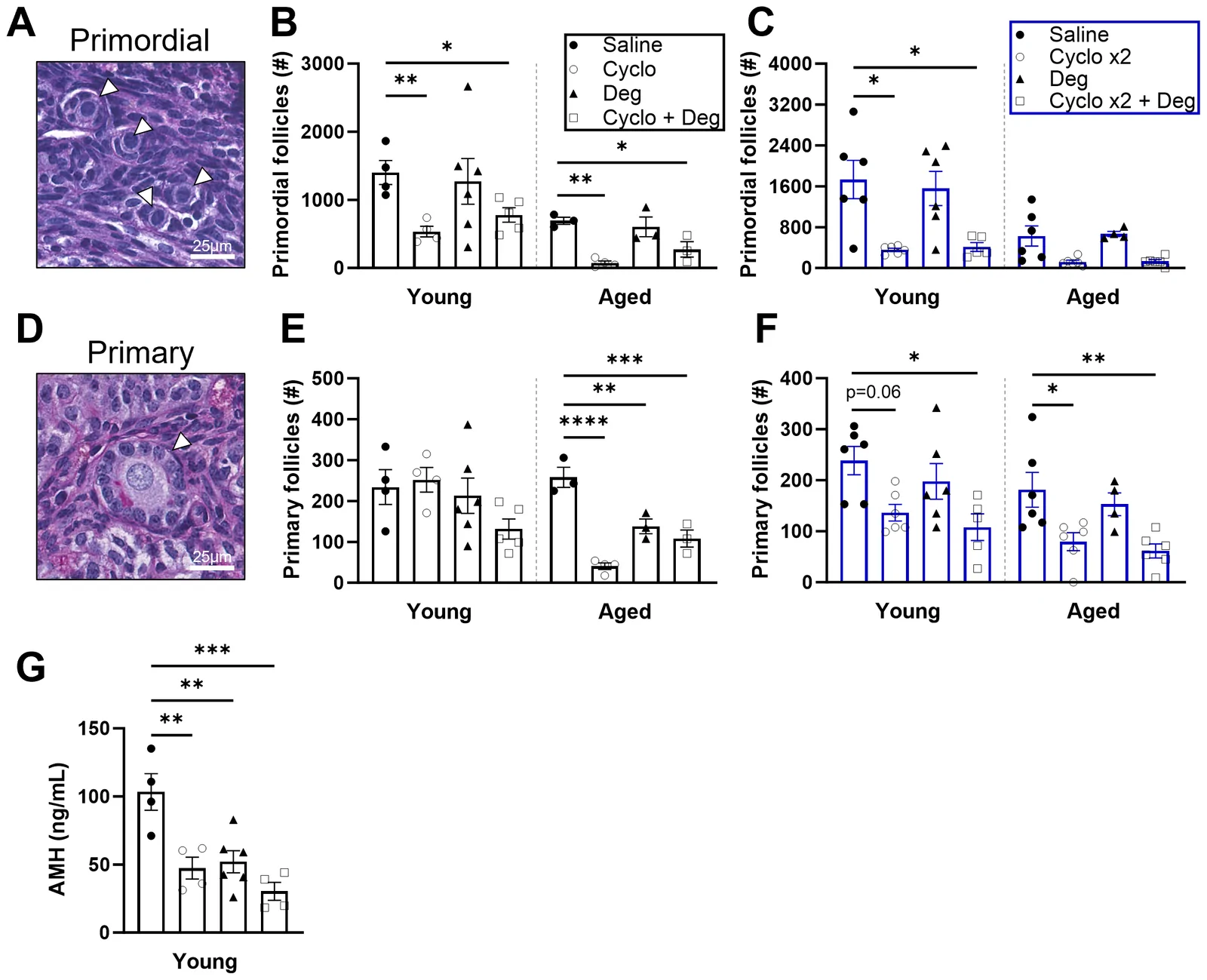
Primordial and primary follicle numbers and serum AMH concentrations following treatment. **(A)** Representative PAS-stained ovarian section showing healthy primordial follicles (arrowheads). Scale bar: 25 µm. **(B, C)** Quantification of healthy primordial follicles in reproductively young and aged tumour-bearing mice from the single-dose and two-dose cyclophosphamide cohorts, respectively. **(D)** Representative PAS-stained ovarian section showing a healthy primary follicle (arrowhead). Scale bar: 25 µm. **(E, F)** Quantification of healthy primary follicles in the single-dose and two-dose cohorts, respectively. Follicles were enumerated using the direct counting method, with raw counts adjusted for the histological sampling interval. **(G)** Serum anti-Müllerian hormone (AMH) concentrations in reproductively young tumour-bearing mice from the single-dose cohort, measured by enzyme-linked immunosorbent assay. Each data point represents one independent animal. Sample sizes for follicle enumeration in the single-dose cohort were n = 5, 4, 6 and 5 for young saline, cyclophosphamide, degarelix and combination groups, respectively; corresponding aged groups comprised n = 4, 4, 3 and 3. In the two-dose cohort, follicle enumeration comprised n = 6, 6, 6 and 5 in young mice and n = 6, 6, 4 and 6 in aged mice. Serum AMH measurements comprised n = 5, 4, 6 and 4 for young saline, cyclophosphamide, degarelix and combination groups, respectively. Data are presented as mean ± SEM. All data were tested for normality using Shapiro-Wilk test and homogeneity variance using Brown-Forsythe test. All data were analysed using a one-way ANOVA with Šidák’s multiple-comparisons test, Brown-Forsythe and Welch’s ANOVA with Dunnett’s T3 or Kruskal-Wallis test with Dunn’s multiple comparisons. *p ≤ 0.05, **p ≤ 0.01 ***p ≤ 0.001, ****p ≤ 0.0001.

### 3.4 Serum AMH concentrations following single-dose treatment

Serum AMH concentration was measured in the young single-dose cyclophosphamide cohort. Compared with controls, AMH levels were significantly reduced by 54% following cyclophosphamide alone (p=0.005), 50% following degarelix alone (p=0.005) and 71% following co-treatment (p=0.0005) (**Figure 3G**).

### 3.5 Impacts of cyclophosphamide with or without degarelix on secondary and antral follicle numbers

In aged tumour-bearing mice, secondary follicle number was significantly reduced compared with controls by 72% (p=0.02) following single-dose and 46% (p=0.02) following two-dose cyclophosphamide; no changes were observed in young mice (**Figures 4A–C**). Comparable depletion was observed in the cyclophosphamide and degarelix co-treated aged mice from single-dose and two-dose cyclophosphamide cohorts (p=0.04 & p=0.0007) (**Figures 4A–C**). Antral follicle numbers did not differ between treatment groups in either age group in the single dose cyclophosphamide cohort (**Figure 4D**). Following two doses of cyclophosphamide, antral follicle number in young mice was reduced compared with controls following degarelix alone (p=0.0002) and cyclophosphamide and degarelix co-treatment (p=0.01) (**Figure 4E**). In aged mice, antral follicle number in co-treated mice was reduced compared to control (p=0.03) and two doses of cyclophosphamide alone (p=0.02) (**Figure 4E**).

**Figure 4:**
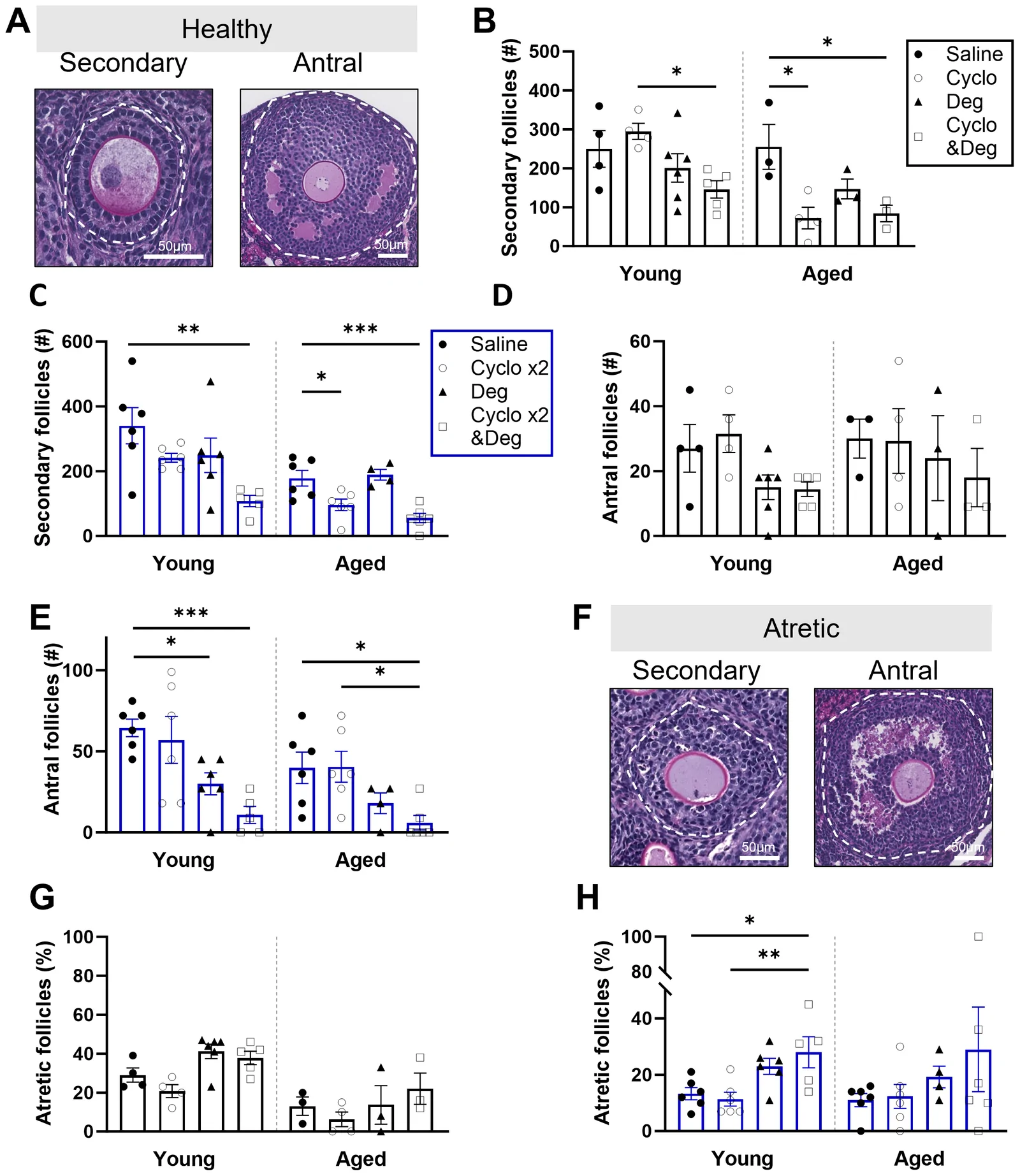
Secondary and antral follicle numbers and growing follicle atresia following treatment. **(A)** Representative PAS-stained ovarian sections showing healthy secondary and antral follicles, outlined with dashed lines. Scale bars: 50 µm. **(B, C)** Quantification of healthy secondary follicles in reproductively young and aged tumour-bearing mice from the single-dose and two-dose cyclophosphamide cohorts, respectively. **(D, E)** Quantification of healthy antral follicles in the corresponding treatment cohorts. **(F)** Representative PAS-stained ovarian sections showing atretic secondary and antral follicles, outlined with dashed lines. Scale bars: 50 µm. **(G, H)** Proportion of atretic growing follicles in the single-dose and two-dose cohorts, respectively. The proportion was calculated by dividing the number of atretic secondary and antral follicles by the total number of healthy and atretic secondary and antral follicles. Each data point represents one independent animal. Sample sizes for the single-dose cohort were n = 5, 4, 6 and 5 for young saline, cyclophosphamide, degarelix and combination groups, respectively; corresponding aged groups comprised n = 4, 4, 3 and 3. Sample sizes for the two-dose cohort were n = 6, 6, 6 and 5 for young mice and n = 6, 6, 4 and 6 for aged mice. Data are presented as mean ± SEM. All data were tested for normality using Shapiro-Wilk test and homogeneity variance using Brown-Forsythe test. All data were analysed using a one-way ANOVA with Šidák’s multiple-comparisons test, Brown-Forsythe and Welch’s ANOVA with Dunnett’s T3 or Kruskal-Wallis test with Dunn’s multiple comparisons. *p ≤ 0.05, **p ≤ 0.01 ***p ≤ 0.001.

### 3.6 Growing follicle atresia following treatment

The proportion of atretic follicles was similar between treatment groups in either age group in the single-dose cyclophosphamide cohort (**Figure 4F**, **G**). The proportion of atretic follicles was significantly higher in young mice co-treated with degarelix and two doses of cyclophosphamide, compared to control (p=0.02) and two dose cyclophosphamide-treated young mice (p=0.009) (**Figure 4H**). No statistically significant differences were detected between treatment groups in aged mice in the two-dose cyclophosphamide cohort (**Figure 4H**).

## 4 Discussion

This study investigated whether GnRH antagonist-induced ovarian suppression can protect the ovary from cyclophosphamide-induced damage *in vivo* in a tumour-bearing mouse model. In doing so, it addresses the less well-characterised effects of GnRH antagonists, compared with the more extensively studied agonists, and provides mechanistic insight into ovarian suppression strategies during cyclophosphamide chemotherapy. Degarelix effectively modelled ovarian suppression, as evidenced by the absence of corpora lutea and marked reduction in ovarian volume. Corpora lutea persisted in tumour-bearing mice administered with single and two-dose cyclophosphamide alone, indicating ovulatory activity despite exposure to chemotherapy. We demonstrate that GnRH antagonist co-treatment fails to prevent cyclophosphamide induced depletion of the ovarian reserve, reduced serum AMH concentrations, and growing follicle loss. Furthermore, co-treatment significantly elevated ovarian fibrosis in young tumour-bearing mice, demonstrating the potential for GnRH antagonists to introduce additional and potentially damaging ovarian structural changes, independent of any protective benefit. Our study evaluated GnRH analogue co-treatment in tumour-bearing mice across two reproductive age groups and two cyclophosphamide dosing regimens. This design enabled comparisons of age-dependent and treatment-dependent ovarian responses within a single experimental model.

Interestingly, there were no significant differences in the number of corpora lutea in mice treated with cyclophosphamide compared to control mice, although other studies utilising non-tumour-bearing models have reported fewer corpora lutea following cyclophosphamide treatment (4, 50, 51). A key strength of our study is the use of tumour-bearing mice, which more closely reflects the clinical context of chemotherapy treatment and biodistribution in cancer patients. Given the limited data on how tumours affect ovarian function, it is possible that tumour burden modified or masked some cyclophosphamide effects, however, corpora lutea number we observed broadly align with those reported in mice of equivalent strain and age (42). In contrast, degarelix alone or degarelix co-treatment with cyclophosphamide resulted in complete loss of corpora lutea, indicating effective gonadotropin suppression and ovulation blockade in tumour-bearing mice. Most studies evaluating GnRH analogue co-treatment during chemotherapy have not assessed corpora lutea, making this another strength of our study. Of the few studies that have, Lemos et al. reported no change in corpora lutea number in Wistar rats treated with cetrorelix alone or with cyclophosphamide, yet, the study only examined 4 ovarian sections per animal. Kovacs et al. compared effects of GnRH antagonist, MI-1544 and GnRH agonist, ovurelin (52). Agonist treated mice still had persistent corpora lutea, whereas antagonist treated mice lacked corpora lutea 22 days after treatment.

Degarelix also led to a profound decrease in ovarian volume, validating ovarian suppression. Ovarian volume is used in clinical settings as a measure of the ovarian reserve and has been shown to correlate with primordial follicle number but is likely more representative of larger ovarian components such as corpora lutea (47, 48). Our findings align with human data reporting reduced ovarian volume measured by ultrasonography following 14 consecutive days of GnRH agonist treatment during assisted reproductive technology (53).

It is well-established that cyclophosphamide is ovotoxic, with single doses between 20–500 mg/kg causing irreversible ovarian reserve depletion in mice (4, 54–59). At 100 mg/kg, single and two-dose cyclophosphamide consistently and significantly depleted the ovarian reserve in young and aged tumour-bearing mice. Degarelix co-treatment did not prevent this reduction in either age group or either regimen. Furthermore, degarelix did not restore the reduction in serum AMH levels after cyclophosphamide treatment. The failure of ovarian suppression to protect the ovarian reserve is mechanistically consistent with the gonadotropin independence of primordial follicle maintenance and activation (60). In contrast, one study using an immunodeficient MCF-7 tumour-bearing mouse model reported that co-treatment with goserelin, a GnRH agonist, protected AMH levels and ovarian reserve during 200 mg/kg cyclophosphamide therapy (40). However, the independent effects of goserelin alone could not be observed as the study lacked goserelin alone or cyclophosphamide alone groups. MCF-7 tumours are oestrogen receptor-positive, and GnRH analogues may exert direct antiproliferative effects on these tumours (61). Consequently, differences in tumour burden could have influenced the reported ovarian outcomes, making it difficult to attribute the findings solely to gonadotrophin suppression. Our findings align with studies demonstrating that GnRH analogues do not reliably protect the ovarian reserve from cyclophosphamide and cisplatin chemotherapy (38, 39, 41, 62, 63). Parlakgumus et al. reported GnRH agonist leuprolide acetate (1 mg/kg subcutaneous) and cetrorelix acetate (0.1 mg/kg intraperitoneal) were unable to protect the ovarian reserve from a single intraperitoneal dose of 75 mg/kg cyclophosphamide in Wistar rats (38). Moreover, cetrorelix depleted the ovarian reserve in absence of cyclophosphamide. Similarly, Danforth et al. observed ovarian reserve depletion in C57BL/6 mice treated with cetrorelix (41). Although we did not detect impacts of degarelix alone on the ovarian reserve, we observed decreases in AMH in young mice treated with degarelix alone. The potential for GnRH analogues themselves to exert ovarian toxicity, together with the persistence of ovarian reserve depletion in degarelix co-treated mice, raises concerns of their use in clinical practice to protect the ovary from chemotherapy-mediated damage.

Ovarian fibrosis is a pathological change generally associated with impaired ovarian function. Our data align with previous work demonstrating cyclophosphamide induces ovarian fibrosis in young tumour-bearing mice (17, 18, 64). In our model, degarelix co-treatment with single dose cyclophosphamide led to greater ovarian fibrosis than cyclophosphamide alone. These findings contrast with Abdelzaher et al., which used Masson’s trichrome staining to assess fibrosis in Wistar rats treated with cyclophosphamide (200 mg/kg bolus, then 8 mg/kg/day for 14 days) with or without GnRH agonist triptorelin (65). They reported widespread collagen deposition after cyclophosphamide alone, which was attenuated by triptorelin. These differences may reflect differences in collagen staining methods or the more intensive cyclophosphamide dosing protocol applied in the previous study.

In our model, mice received degarelix followed by cyclophosphamide within one hour, similar to regimens by Lemos et al. and Parlakgumus et al., in which cetrorelix was administered one hour before cyclophosphamide (38, 66). GnRH antagonists suppressed serum LH concentrations by 51-84% and FSH concentrations by 17-42% within 8–24 hours after administration (66, 67), whereas GnRH agonists require ~14 days to achieve pituitary suppression after an initial stimulatory effect (30). Although GnRH antagonists are considered to act ‘immediately’, suppression still develops over several hours and therefore may have been incomplete prior to cyclophosphamide administration in our study. This timing and dosing frequency could have limited potential ovarian protective effects of degarelix, particularly as cyclophosphamide induces ovarian damage rapidly (4, 62, 68).

## 5 Conclusion

Degarelix-induced ovarian suppression did not protect the ovarian reserve from cyclophosphamide-induced depletion in reproductively young or aged tumour bearing mice following either single or two-dose regimens. Co-treatment was additionally associated with increased ovarian fibrosis and follicle atresia and reduced antral follicle number and serum AMH concentrations in young mice. Ovarian suppression alone was therefore not sufficient to preserve the ovarian reserve in this model and may itself have contributed to ovarian injury. These findings should be interpreted in the context of two main limitations. First, the use of a tumour volume endpoint introduced variability in collection age and does not account for the potential impact of tumour burden between treatment groups. Furthermore, ovarian suppression was achieved using degarelix, a GnRH antagonist, whereas GnRH agonists are commonly used clinically. Future studies should extend upon these findings and investigate functional reproductive outcomes through natural breeding or *in-vitro* fertilisation using oocytes collected following treatment cessation and recovery. Furthermore, whether the stromal changes identified in this study persist beyond treatment remains unknown and warrants investigation. These findings highlight the need for further preclinical investigation of clinically used GnRH agonists and antagonists across chemotherapy regimens and age groups, incorporating reproductive outcomes in tumour-bearing models before extrapolating their potential ovarian protective effects to clinical settings.

## Data Availability

The original contributions presented in the study are included in the article/**Supplementary Material**. Further inquiries can be directed to the corresponding authors.

## Supplementary material

Supplementary Figure 1 Representative ovarian stromal morphology following degarelix and cyclophosphamide treatment. Representative PAS-stained ovarian sections showing stromal morphology in reproductively young and aged tumour-bearing mice treated with saline, cyclophosphamide, degarelix or combination treatment. Images are presented for the single-dose and two-dose cyclophosphamide cohorts. White asterisks indicate zona pellucida remnants. Scale bar: 50 µm.

Supplementary Table 1 Allocation of mice to treatment groups and analyses. Values indicate the number of mice per treatment group in the single- and two-dose cyclophosphamide cohorts, and the analyses to which each sample contributed. Mice excluded prior to analysis are marked with an asterisk; reasons for exclusion are given below the table.
